# Exploring stakeholder perceptions of implementing a pharmacist-led anticoagulation clinic in primary care settings: a cross-sectional study

**DOI:** 10.1080/20523211.2024.2395529

**Published:** 2024-09-06

**Authors:** Safaa Alshihab, Mohamed Izham Mohamed Ibrahim, Muhammad Abdul Hadi, Abdullah Syed, Abdul Rahman Arabi, Awad Al-Qahtani, Hanan Al Mujalli, Ihsan Rafie, Mohamed Gaith Al-Kuwari, Mujeeb Kandy, Manal Al-Zaidan

**Affiliations:** a Clinical Pharmacy and Practice Department, College of Pharmacy, QU Health, Qatar University, Doha, Qatar; b Primary Health Care Corporation, Doha, Qatar; c Heart Hospital, Hamad Medical Corporation, Doha, Qatar; d College of Medicine, Qatar University, Doha, Qatar

**Keywords:** Anticoagulation, primary care, pharmacist-led, implementation research

## Abstract

**Background:**

Anticoagulation therapy is crucial for managing various cardiovascular and thrombotic conditions; however, optimal delivery remains challenging in primary care. Pharmacist-led anticoagulation services have emerged as a potential strategy for enhancing patient care and outcomes in such settings. Understanding the perspectives of key stakeholders is critical for successful implementation.

**Objectives:**

This study aimed to explore the perceptions of key stakeholders involved in the implementation of pharmacist-led anticoagulation clinics in primary care settings.

**Methods:**

A cross-sectional study was conducted using structured, pilot-tested questionnaires between August and October 2023. Patients receiving warfarin, pharmacists, and physicians working across various primary healthcare centres were invited to complete an online survey. Each group of stakeholders had individualised questionnaires to assess their perceptions and expectations with regard to developing pharmacist-led anticoagulation clinics in primary care. Descriptive statistics were used to analyze the data.

**Results:**

The response rates for the survey were 29.4% for physicians, 10.4% for patients, and 48.6% for pharmacists. Participants expressed positive perceptions toward pharmacist-led anticoagulation clinics, acknowledging benefits such as improved access to care, enhanced medication management, and increased patient education. The respondents expressed confidence in the expertise and skills of pharmacists in this role. However, healthcare providers strongly agree that pharmacists should receive additional training specific to anticoagulation management. Establishing standardised protocols and fostering interprofessional collaboration were identified as the main facilitators for practical implementation.

**Conclusions:**

Broad support exists for pharmacist-led anticoagulation clinics in primary care, though additional pharmacist training and accountability concerns need to be addressed for successful implementation.

## Introduction

Anticoagulation therapy plays a pivotal role in the management of thromboembolic disorders. However, balancing the risks of bleeding and thromboembolic complications presents a significant challenge. A systematic review of 12 randomised controlled trials found that major bleeding occurred in 4% of patients treated with direct oral anticoagulants (DOACs) and in 4.64% of patients treated with vitamin K antagonists (VKAs). Additionally, fatal bleeding occurred in 0.30% of patients treated with DOACs and in 0.52% of patients treated with VKAs (Chai-Adisaksopha et al., 2014).

Specialised anticoagulation clinics have emerged as a promising solution, demonstrating fewer bleeding and thromboembolic events than standard care. Notably, these clinics offer a cost-effective alternative that is primarily driven by reduced hospitalisations and emergency visits related to anticoagulation complications (Garay et al., 2022; Hou et al., 2018; Rudd & Dier, 2010). A previous study showed that dedicated anticoagulation services led to annual net savings of more than $240,000 per 100 patients compared to routine physical care (Aziz et al., 2011).

Pharmacist-led anticoagulation models have demonstrated superior anticoagulation control and positive outcomes compared with other models (Hou et al., 2018). The extent of pharmacist involvement varies according to country and organisational regulations but typically includes tasks such as INR monitoring, dosage recommendations, patient education, and follow-up scheduling (Hou et al., 2018; Raphael, 2020).

Recently, there has been a widespread shift in the movement of anticoagulation care from secondary care to primary care settings, where pharmacists play a crucial role in managing anticoagulation therapy. (Ingram et al., 2018; Shaw et al., 2014). This transition aims to improve patient accessibility, alleviate the workload of general practitioners, and reduce the burden on secondary and tertiary care settings. These services have shown improved anticoagulation control, decreased rates of minor bleeding events, and enhanced quality of life compared with standard care (Falamić et al., 2018; Harper et al., 2015; Harrison et al., 2015; Ingram et al., 2018; Woodill & Bodnar, 2020).

Different anticoagulation clinic models have been developed in Qatar in hospital settings (Alhmoud et al., 2021; Elewa et al., 2016a; Elewa et al., 2016b), showing better control and higher patient satisfaction than physician-managed clinics (Abdallah et al., 2021; Elewa et al., 2016b). However, no such pharmacist-led anticoagulation care is available in primary care. This study was part of a broader effort to evaluate the scope for implementing a specialised pharmacist-led anticoagulation clinic in the country’s leading public primary care provider; the Primary Health Care Corporation (PHCC) (Al Kuwari et al., 2022). The initial phase focused on a comprehensive evaluation of warfarin management in the PHCC centres, revealing suboptimal control and various challenges in anticoagulation management in primary care settings (Alshihab et al., 2024). Consequently, it is recommended to implement dedicated anticoagulation clinics across the PHCC health centres to address these challenges.

Although existing evidence primarily examines the role of community pharmacists in anticoagulation services within community pharmacies, further evaluation of stakeholder perspectives on pharmacist-managed anticoagulation clinics in primary healthcare centres, especially in the Middle East, is necessary. Incorporating stakeholders’ viewpoints and patient engagement in service development is essential for fostering patient-centered care, addressing the needs and expectations of all parties involved, and ultimately improving health outcomes (Potthoff et al., 2023; Prior & Campbell, 2018). Thus, the second phase was conducted to explore stakeholders’ perceptions about the implementation of specialised anticoagulation clinics in the PHCC through a mixed-methods design. The qualitative study was conducted with healthcare administrators and will be reported separately. This present study’s specific objective was to assess the perceptions of key stakeholders (i.e. patients on warfarin, pharmacists, and physicians) involved in implementing pharmacist-led anticoagulation clinics in primary care settings.

## Methods

### Study design

A cross-sectional survey was undertaken among patients, pharmacists, and physicians using SurveyMonkey®. Online surveys offer several advantages. First, they enabled us to gather data efficiently from a geographically diverse population within a designated timeframe. Second, the anonymity provided by the online format encouraged honest responses, which was particularly important considering the inclusion of institutional employees (Critical Thinking in Clinical Research: Applied Theory and Practice Using Case Studies, 2018; Fowler, 2009).

### Study settings

The study was conducted at the PHCC between August and October 2023. The PHCC operates a network of 31 health centres, offering accessible and high-quality services that emphasise preventive care, health promotion, and the management of common health issues. This focus on preventative measures contributes to the overall health and well-being of the population (Al Kuwari et al., 2022).

### Population and sampling

The surveys were sent to all patients on warfarin managed at the PHCC between January 2018 and July 2023, as well as all pharmacists and physicians working at the PHCC during the study period (August 2023). The surveys were shared with the entire population considering multiple factors such as a relatively small and well-defined population, negligible costs, and a high level of precision (Draugalis & Plaza, 2009; Martínez-Mesa et al., 2016). Therefore, a universal sampling technique was used (Fowler, 2009; Thygesen & Ersbøll, 2014).

### Questionnaire design and data collection

Surveys were developed following a comprehensive examination of the published literature, focusing on anticoagulation services provided by pharmacists (Harrison et al., 2015; Shaw et al., 2014). The patient survey comprised 31 questions and was divided into four sections. The first section collected sociodemographic data, including gender, age, nationality, education level, marital status, and average monthly household income. Section 2 focused on the patient’s clinical characteristics, such as indication and duration of warfarin use. The third section explored patients’ perceptions regarding the implementation of specialised anticoagulation services in primary care settings. The fourth section was designed to explore patients’ perceptions about the provision of anticoagulation services by pharmacists. Respondents were required to rank their level of agreement or disagreement with the items on a 5-point Likert scale in section 3 and 4.

The healthcare providers’ surveys were divided into four sections, including 34 and 42 questions for the pharmacists and physicians surveys, respectively. The first section included questions about the demographic characteristics of the respondents and their level of participation in managing patients on warfarin. The second section explored the participants’ perceptions of implementing a specialised anticoagulation clinic in a primary care setting. Section 3 was designed to explore the participants’ perceptions regarding the management of these clinics by a clinical pharmacist. The last section discussed the potential barriers and facilitators of implementing a pharmacist-led anticoagulation clinic in the PHCC. Respondents were required to rank their level of agreement or disagreement with the items on a 5-point Likert scale in sections 2, 3, and 4.

The questionnaire was developed in English and translated into Arabic following a rigorous translation process. This process involved forward and backward translation by two bilingual researchers to ensure the questionnaires’ accuracy (Tsang et al., 2017).

Face validity was used to assess the validity of the survey. Face validity involves seeking expert opinions on whether an instrument effectively measures the intended concept (Roberta & Alison, 2015). A panel of experts in social pharmacy and administration research from Qatar University and PHCC was engaged to assess and refine the generated items, ensuring their validity and alignment with the study objectives. A pilot phase was conducted to determine the comprehensibility and clarity of the survey instruments. During this stage, the surveys were sent to a select group of three patients, 17 pharmacists, and 5 physicians. The objectives of the pilot process were explicitly communicated to them, highlighting our intention to refine the content of the survey. Participants’ feedback was collected and analyzed. These insights were used to refine the content and structure of the survey.

Patient contact numbers were retrieved from the electronic health record. Patients were reached using an SMS text message. The message contained a brief statement of the study objective and a link to a bilingual (Arabic/English) online survey. Two reminders were sent each week apart. A list of pharmacists’ and physicians’ official emails was obtained from the Human Resources Department of the PHCC. Healthcare providers were contacted via their official emails. The survey was developed in English to include the study background, consent form, and survey questions.

### Data analysis

The survey responses were analyzed using descriptive statistics, including frequencies and proportions. The data were analyzed using the statistical software, SPSS (Version 28.0).

## Results

### Physicians

Eight hundred thirty-eight physicians were invited to participate in this study. Two hundred and forty-six responded to the survey, resulting in a response rate of 29.4%. Of the returned surveys, 41 cases (16.6%) were excluded from the analysis because of incomplete surveys, and an additional 8 cases (3.3%) were excluded because the participants belonged to a different specialty than our intended target group. Therefore, 197 participants were included in the analysis. Most participants were male (55.8%, n = 110) and aged between 45 and 54 years (41.1%, n = 81). Approximately 67% of the participants were family physicians (n = 133).

Most respondents (55.8%, n = 110) reported that they provided care for patients requiring warfarin 1–5 times per month, and 37.1% (n = 73) reported that they did not provide regular care for patients requiring warfarin. Fifty-seven physicians (28%) indicated they were not confident or slightly confident in providing warfarin management. Meanwhile, 52.8% (n = 104) of the participants were fairly to completely confident in providing care to patients on warfarin.

More than two-thirds of the physicians agreed (44.2%, n = 87) or strongly agreed (32%, n = 63) that anticoagulation clinics in primary care settings would be as efficient as hospital-based clinics. Approximately 66% agreed (40.1%, n = 79) to strongly agree (25.9%, n = 51) that additional management of anticoagulation therapy in primary care settings is needed. Most physicians agreed or strongly agreed that providing anticoagulation services in primary care settings would be more convenient for patients (66.5%, n = 131), facilitate appointment scheduling (71.6%, n = 141), improve patient education (75.1%, n = 148), and save patient time (71.6%, n = 141). Physicians indicated that they would be supported in managing their patients in a specialised anticoagulation clinic in primary care, with 70.6% (n = 139) of them either strongly agreeing or agreeing. The majority of physicians strongly agreed or agreed that implementing the clinic in primary care would reduce the fragmented care provided for patients on anticoagulation (67.0%, n = 132) and pressure on other healthcare settings (72.6%, n = 143). Most physicians (67.5%, n = 133) indicated that they might be up to date on their patients’ issues, and the majority (34.5%, n = 68) disagreed that they would be less aware of their patients’ conditions if they enrolled in the clinic. More than half of the respondents agreed that developing a primary care clinic could enhance patient care documentation for patients on warfarin (52.3%, n = 103). Table 1 presents the physicians’ perceptions regarding the implementation of a specialised anticoagulation clinic in primary care settings.

**Table 1.** Physicians’ perceptions toward implementing a pharmacist-led anticoagulation clinic in primary care settings (N = 197).

Item	Strongly agree n (%)	Agree n (%)	Neither agree nor disagree n (%)	Disagree (n,%)	Strongly disagree (n,%)
Patients taking warfarin would benefit from this service as efficiently as hospital-provided services	63 (32)	87 (44.2)	21 (10.7)	8 (4.1)	2 (1.0)
More management of anticoagulation agents should be undertaken in primary care settings	51 (25.9)	79 (40.1)	32 (16.2)	13 (6.6)	6 (3.0)
It is more convenient for patients to manage their warfarin in primary healthcare settings	55 (27.9)	76 (38.6)	31 (15.7)	13 (6.6)	6 (3.0)
Specialised anticoagulation clinics in primary care settings will optimise patient education on warfarin	67 (34.0)	81 (41.1)	21 (10.7)	9 (4.6)	3 (1.5)
The clinic appointments will be more at ease in a primary care setting	58 (29.4)	83 (42.1)	23 (11.7)	13 (6.6)	4 (2.0)
Providing anticoagulation services in primary care settings would save time for patients taking warfarin	66 (33.5)	75 (38.1)	30 (15.2)	7 (3.6)	3 (1.5)
Physicians would be supportive of managing patients on warfarin in a specialised anticoagulation clinic in primary care setting	48 (24.4)	91 (46.2)	29 (14.7)	9 (4.6)	4 (2.0)
Providing anticoagulation management services in primary care settings will reduce the fragmented care provided for patients on warfarin	48 (24.4)	84 (42.6)	40 (20.3)	8 (4.1)	1 (0.5)
Providing anticoagulation management services in primary care settings may alleviate the pressures on secondary and tertiary care facilities	57 (28.9)	86 (43.7)	28 (14.2)	7 (3.6)	3 (1.5)
Since my patients have been managed in the anticoagulation clinic, their warfarin treatment could be less well controlled	12 (6.1)	41 (20.8)	67 (34.0)	53 (26.9)	8 (4.1)
I will be less aware of changes in my patient’s medical condition if they enrol in the clinic	8 (4.1)	37 (18.8)	58 (29.4)	68 (34.5)	10 (5.1)
I feel that I may be kept up to date on changes in therapy and other relevant issues for my patients	33 (16.8)	100 (50.8)	44 (22.3)	4 (2.0)	0 (0.0)
I believe that developing an anticoagulation clinic in primary care centres will improve care documentation for patients on warfarin	31 (15.7)	103 (52.3)	29 (14.7)	7 (3.6)	0 (0.0)
Clinical pharmacists are in an excellent position to manage warfarin effectively	27 (13.7)	97 (49.2)	42 (21.3)	10 (5.1)	2 (1.0)
I am confident that the clinical pharmacist can safely manage my patients’ warfarin treatment	27 (13.7)	94 (47.7)	46 (23.4)	9 (4.6)	2 (1.0)
Clinical pharmacists cannot initiate warfarin therapy as efficiently as hospital-based physicians	32 (16.2)	73 (37.1)	39 (19.8)	33 (16.8)	1 (0.5)
Pharmacist-led anticoagulation clinics will improve warfarin adherence for patients who are enrolled	29 (14.7)	98 (49.7)	47 (23.9)	3 (1.5)	1 (0.5)
Pharmacists-led anticoagulation clinics are able to provide good education to patients on warfarin	46 (23.4)	108 (54.8)	22 (11.2)	2 (1.0)	0 (0.0)
Clinical pharmacists need additional training in order to monitor patients on warfarin	54 (27.4)	89 (45.2)	30 (15.2)	3 (1.5)	2 (1.0)
Close monitoring of warfarin by the clinical pharmacist will decrease the likelihood of adverse events in my patients	45 (22.8)	108 (54.8)	23 (11.7)	2 (1.0)	0 (0.0)
I would be uncomfortable referring my patients to a pharmacist-led anticoagulation clinic in primary care settings if I were to be held responsible if something were to go wrong	25 (12.7)	54 (27.4)	51 (25.9)	41 (20.8)	7 (3.6)
Patients on warfarin will accept the clinical pharmacist’s recommendations provided in the anticoagulation clinic	21 (10.7)	85 (43.1)	57 (28.9)	7 (3.6)	0 (0.0)
Patients may have doubts about the quality of care they receive from pharmacists-managed anticoagulation clinics	7 (3.6)	58 (29.4)	64 (32.5)	38 (19.3)	3 (1.5)
Patients would prefer to have their warfarin anticoagulation managed by their physician rather than a clinical pharmacist	16 (8.1)	65 (33.0)	66 (33.5)	21 (10.7)	2 (1.0)
Anticoagulation management by a clinical pharmacist will decrease the number of questions I receive from patients about warfarin	21 (10.7)	96 (48.7)	48 (24.4)	5 (2.5)	0 (0.0)
Anticoagulation management by a clinical pharmacist will decrease the amount of time I spend on anticoagulation issues for my patients	28 (14.2)	97 (49.2)	41 (20.8)	3 (1.5)	1 (0.5)
My patients’ enrolment in the anticoagulation clinic may affect my relationship with them	9 (4.6)	44 (22.3)	45 (22.8)	55 (27.9)	17 (8.6)
Having my patients enrolled in the anticoagulation clinic may improve my relationship with the pharmacist	29 (14.7)	99 (50.3)	39 (19.8)	2 (1.0)	1 (0.5)
Physicians will have a smooth and efficient referral process for patients on warfarin, directing them to the anticoagulation clinic as needed	23 (11.7)	111 (56.3)	30 (15.2)	6 (3.0)	0 (0.0)
I believe that a lack of information sharing between physicians and the clinical pharmacist could affect warfarin management	40 (20.3)	95 (48.2)	30 (15.2)	5 (2.5)	0 (0.0)
Well-established protocol for managing patients on warfarin will improve the clinic's efficacy	62 (31.5)	90 (45.7)	17 (8.6)	0 (0.0)	1 (0.5)
The immediate availability of INR results through point-of-care devices, and immediate dose adjustments will contribute to lower stress reported across patients	65 (33.0)	83 (42.1)	22 (11.2)	0 (0.0)	0 (0.0)
Appropriate cooperation with healthcare professionals, peers, or expert support, may facilitate implement an anticoagulation clinic managed by a pharmacist	53 (26.9)	88 (44.7)	28 (14.2)	1 (0.5)	0 (0.0)

*The percentages shown in the results do not total 100% as some participants chose not to answer certain questions

In assessing physicians’ perceptions of implementing a pharmacist-led anticoagulation model, more than 60% of the respondents agreed or strongly agreed that clinical pharmacists are able to manage warfarin therapy effectively (62.9%, n = 124) and ensure its safe administration (61.4%, n = 121). However, most patients (53.3%, n = 105) indicated that clinical pharmacists are not as efficient as hospital-based physicians in initiating warfarin therapy. Most respondents agreed that a pharmacist-led anticoagulation clinic would decrease the occurrence of warfarin-related adverse events (54.8%, n = 108) and enhance patient adherence to anticoagulation therapy (49.7%, n = 98). A substantial proportion of the respondents (54.8%, n = 108) agreed that a clinical pharmacist could properly educate patients about warfarin. However, more than 72% (n = 143) of physicians agreed or strongly agreed that clinical pharmacists require additional training to provide effective patient monitoring. Concerns were expressed about accountability, with 40.1% (n = 79) of the respondents either agreeing or strongly agreeing that they would be uncomfortable referring patients to the anticoagulation clinic if they were held responsible for potential issues. However, most respondents did not agree or disagree that patients may have concerns about the quality of care provided by the clinical pharmacist (32.5%, n = 64) and prefer to have their warfarin anticoagulation therapy overseen by their physicians (41.1%, n = 81). Most physicians agreed that having a clinical pharmacist manage anticoagulation would reduce the time spent on anticoagulation issues for patients (49.2%, n = 97) and the number of patient inquiries regarding warfarin (48.7%, n = 96). Most physicians disagreed (27.9%, n = 55) or strongly disagreed (8.6%, n = 17) that having their patients managed in an anticoagulation clinic could affect their relationships. Moreover, approximately half of the respondents (50.3%, n = 99) agreed that implementing a clinic could enhance their relationships with pharmacists.

The last section of the survey assessed physicians’ perceptions of facilitators and barriers to implementing a pharmacist-led anticoagulation clinic in a primary care setting. Most respondents agreed that a well-established guideline for managing patients on warfarin (45.7%, n = 90) and proper collaboration among healthcare providers could facilitate the clinic’s implementation and improve its efficacy (44.7%, n = 88). They believed that instant INR values obtained from the Point of Care (POC) and subsequent dose adjustment would reduce the stress experienced by patients (42.1%, n = 83). The majority agreed (48.2%, n = 95) or strongly agreed (20.3%, n = 40) that a lack of shared information between physicians and pharmacists could hinder clinic development. A notable proportion of respondents agreed (56.3%, n = 111) to strongly agree (11.7%, n = 23) that they would have a hassle-free referral system.

### Pharmacists

Three hundred and eighty-eight pharmacists were invited to participate in this study. A total of 188 respondents responded to the survey, representing a response rate of 48.6%. Of the returned surveys, 39 (20.7%) were excluded from the analysis because of incomplete surveys. Thus, 149 participants were included in this analysis. The majority of the participants were male (51.7%, n = 77), aged between 35 and 44 years (55.7%, n = 83), and 66.4% held a bachelor’s degree in pharmacy as their highest qualification (n = 99).

Table 2 presents an overview of pharmacists’ perceptions of implementing a pharmacist-led anticoagulation clinic in primary care. A significant percentage of respondents (89.9%, n = 134) indicated their agreement with the potential benefits of providing anticoagulation services in primary care settings, with 53.7% (n = 80) strongly agreeing and 36.2% (n = 54) agreeing. Nearly all participants perceived that providing such services in primary care would be more convenient for patients, with 43.6% (n = 65) strongly agreeing and 38.9% (n = 58) agreeing. Additionally, a significant proportion believed that having an anticoagulation clinic in a primary care setting would facilitate patient education about warfarin, with 56.4% (n = 84) strongly agreeing and 35.6% (n = 53) agreeing. Moreover, a considerable number of pharmacists perceived that appointments at such clinics in primary care settings would be more accessible to patients, with 49.0% (n = 73) strongly agreeing and 38.9% (n = 58) agreeing. A notable majority of the respondents agreed or strongly agreed that the implementation of an anticoagulation clinic in primary care settings would save patient time (90.6%, n = 135), reduce the fragmented care provided to patients (83.9%, n = 125), and alleviate pressure on other healthcare settings (88.6%, n = 132). Pharmacists believed that family physicians would support the provision of specialised anticoagulation services in primary care (80.6%, n = 120). Most respondents strongly agreed that implementing this model in primary care would improve care documentation (44.3%, n = 66).

**Table 2.** Pharmacists’ perceptions toward implementing a pharmacist-led anticoagulation clinic in primary care settings (N = 149).

Item	Strongly agree n (%)	Agree n (%)	Neither agree nor disagree n (%)	Disagree n (%)	Strongly disagree n (%)
Patients taking warfarin would benefit from providing anticoagulation services in primary care settings	80 (53.7)	54 (36.2)	8 (5.4)	7 (4.7)	0 (0.0)
It is more convenient for patients to have anticoagulation management at the primary care centres	65 (43.6)	58 (38.9)	13 (8.7)	13 (8.7)	0 (0.0)
Having an anticoagulation clinic in primary care settings will facilitate educating patients about warfarin	84 (56.4)	53 (35.6)	7 (4.7)	5 (3.4)	0 (0.0)
Appointments at an anticoagulation clinic in a primary care setting will be more convenient for patients	73 (49.0)	58 (38.9)	16 (10.7)	2 (1.3)	0 (0.0)
Providing an anticoagulation service in primary care settings would save time for patients taking warfarin	77 (51.7)	58 (38.9)	12 (8.1)	2 (1.3)	0 (0.0)
Family medicine practitioners would be supportive of managing patients on warfarin in primary care settings	56 (37.6)	64 (43.0)	21 (14.1)	7 (4.7)	1 (0.7)
I believe that providing anticoagulation management services in primary care settings will reduce the fragmented care provided to patients on warfarin	61 (40.9)	64 (43.0)	17 (11.4)	7 (4.7)	0 (0.0)
I believe that providing anticoagulation management services in primary care settings may reduce the pressures on secondary and tertiary care facilities	71 (47.7)	61 (40.9)	13 (8.7)	4 (2.7)	0 (0.0)
Developing an anticoagulation clinic in primary care centres will improve care documentation for patients on warfarin	66 (44.3)	56 (37.6)	12 (8.1)	3 (2.0)	0 (0.0)
Clinical pharmacists are in an excellent position to manage warfarin effectively	70 (47.0)	52 (34.9)	14 (9.4)	7 (4.7)	0 (0.0)
Anticoagulation clinics led by clinical pharmacists will improve warfarin adherence for patients	75 (50.3)	52 (34.9)	12 (8.1)	4 (2.7)	0 (0.0)
Pharmacist-led anticoagulation clinics are able to provide good education to patients on warfarin	82 (55.0)	55 (36.9)	3 (2.0)	3 (2.0)	0 (0.0)
As a direct result of consulting patients for their INR testing, the clinical pharmacist may be able to help them with other aspects of their health care	69 (46.3)	59 (39.6)	14 (9.4)	1 (0.9)	0 (0.0)
I believe that providing anticoagulation management services may improve pharmacists’ relationships with their patients	71 (47.7)	56 (37.6)	13 (8.7)	3 (2.0)	0 (0.0)
I believe that an anticoagulation clinic is an opportunity for pharmacists to use their professional skills in an extended role	84 (56.4)	51 (34.2)	6 (4.0)	1 (0.7)	1 (0.7)
I believe that patients on warfarin will accept the clinical pharmacist’s recommendations provided in the clinic	54 (36.2)	65 (43.6)	14 (9.4)	4 (2.7)	0 (0.0)
Patients may have doubts about the quality of care they may receive from pharmacist-managed anticoagulation clinics	32 (21.5)	38 (25.5)	43 (28.9)	19 (12.8)	5 (3.4)
Family medicine practitioners will be confident in the clinical pharmacist’s ability to manage patients on warfarin	41 (27.5)	74 (49.7)	17 (11.4)	4 (2.7)	1 (0.7)
Family medicine practitioners can allocate extra time and resources to other areas of their practice in the presence of a pharmacist-led anticoagulation clinic in primary care settings	49 (32.9)	65 (43.6)	20 (13.4)	3 (2.0)	0 (0.0)
Pharmacists would need additional training in order to monitor patients on warfarin	79 (53.0)	47 (31.5)	10 (6.7)	1 (0.7)	0 (0.0)
Pharmacists managing patients on warfarin in anticoagulation clinics will improve the pharmacist – physician relationship	72 (48.3)	51 (34.2)	13 (8.7)	1 (0.7)	0 (0.0)
The family medicine practitioner will refer patients on warfarin comfortably to the anticoagulation clinic if needed	50 (33.6)	68 (45.6)	17 (11.4)	1 (0.7)	1 (0.7)
Well-established protocol for managing patients on warfarin will improve the clinic's efficacy	74 (49.7)	55 (36.9)	5 (3.4)	3 (2.0)	0 (0.0)
Lack of support from family medicine practitioners will affect how well the clinic will operate	56 (37.6)	64 (43.0)	14 (9.4)	2 (1.3)	1 (0.7)
I believe that a lack of information sharing between pharmacists and physicians could affect warfarin management	77 (51.7)	50 (33.6)	8 (5.4)	2 (1.3)	0 (0.0)
Appropriate cooperation with health professionals, peers, or expert support, may facilitate the implementation of an anticoagulation clinic managed by a pharmacist	68 (45.6)	58 (38.9)	10 (6.7)	1 (0.7)	0 (0.0)

* The percentages shown in the results do not total 100% as some participants chose not to answer certain questions

The second part of the questionnaire assessed pharmacists’ perceptions of implementing a pharmacist-led model in primary care. Most respondents agreed or strongly agreed (81.9%, n = 122) that a clinical pharmacist is in a good position to manage the clinic effectively. Similarly, most participants perceived that implementing this model would improve patient adherence to anticoagulation therapy (85.2%, n = 127). The majority of survey respondents indicated their agreement or strong agreement that pharmacists possess the capability to offer effective education to patients on warfarin (91.9%, n = 137). However, the majority agreed (53.0%, n = 79) to strongly agree (31.5%, n = 47) that additional training for pharmacists is required to monitor patients on warfarin.

More than two-thirds of the respondents perceived that implementing a pharmacist-led anticoagulation clinic could enable pharmacists to help with other aspects of patients’ healthcare (85.9%, n = 128). A notable proportion of respondents agreed or strongly agreed that implementing this model would improve patient-pharmacist relationships (85.3%, n = 127) and enhance the relationship between pharmacists and physicians (82.5%, n = 123). More than 80% of the pharmacists agreed or strongly agreed (90.6%, n = 135) that implementing this model provides pharmacists with an opportunity to apply their professional expertise in an extended role. The majority perceived that family medicine practitioners would have confidence in a clinical pharmacist’s ability to manage patients taking warfarin (49.7%, n = 74). Most pharmacists agreed that family medicine physicians could allocate extra time to handling other aspects of their practice in the presence of the clinic (43.6%, n = 65). The participants agreed that patients would accept the pharmacists’ recommendations (43.6.2%, n = 65). However, the majority expressed uncertainty (28.9%, n = 43) regarding patients’ perceptions about the quality of service provided by the pharmacist.

In the assessment of pharmacists’ perceptions of facilitators and barriers to establishing a pharmacist-led anticoagulation clinic in primary care settings, most respondents strongly agreed that a well-established protocol (49.7%, n = 74) and effective collaboration with other stakeholders (45.6%, n = 68) would serve as facilitators in the clinic’s implementation. Most respondents agreed that family medicine practitioners could refer their patients to the clinic smoothly (45.6%, n = 68). Most pharmacists agreed that a lack of support from family medicine practitioners could hinder the effective operation of clinics (43.0%, n = 64). Additionally, the majority of participants strongly agreed (51.7%, n = 77) that failure to share information between pharmacists and physicians adversely affects patient management.

### Patients

Survey invitations were sent to 937 patients, of which 97 patients responded, representing a response rate of 10.4%. Twenty-two surveys (22.7%) were excluded from the final analysis because of incomplete responses. Thus, 75 participants were included in the analysis.

The majority of respondents were male (62.7%, n = 47), married (81.3%, n = 61), and aged between 45–54 years (28%, n = 21). The majority of participants were Qataris, comprising more than 30% (n = 23).

The most common indication for warfarin was heart valve replacement, which accounted for 36% of cases (n = 27). Nearly all participants reported being on warfarin for more than 1 year (96%, n = 72). Approximately half of the respondents visited healthcare facilities more than once a month on average for warfarin monitoring (50.7%, n = 38). Twenty-eight percent (n = 21) of the participants had previously been hospitalised for warfarin-related bleeding or thromboembolic events. Table 3 presents an overview of patients’ perceptions regarding the implementation of a specialised anticoagulation clinic in primary care settings.

**Table 3.** Patients’ perception toward implementing a pharmacist-led anticoagulation clinic in primary care settings (N = 75).

Item	Strongly agree n (%)	Agree n (%)	Neither agree nor disagree n (%)	Disagree n (%)	Strongly disagree n (%)
I believe that patients taking warfarin would benefit from implementing anticoagulation clinic in primary healthcare centres	50 (66.7)	15 (20.0)	7 (9.3)	2 (2.7)	1 (1.3)
I feel confident that managing my warfarin therapy in a special clinic in a primary care setting will improve my health	45 (60.0)	19 (25.3)	8 (10.7)	2 (2.7)	1 (1.3)
Managing my warfarin therapy in a special clinic in a primary care setting will improve my adherence to my medicines	40 (53.3)	21 (28.0)	8 (10.7)	5 (6.7)	1 (1.3)
Specialised anticoagulation clinics in primary care settings may increase the time required to discuss my concerns about warfarin	42 (56.0)	16 (21.3)	10 (13.3)	6 (8.0)	1 (1.3)
The clinic appointments will be more at ease in primary healthcare centres	38 (50.7)	14 (18.7)	17 (22.7)	4 (5.3)	2 (2.7)
It is more convenient to have my warfarin management at the primary healthcare centre compared to hospital-based settings	33 (44.0)	22 (29.3)	13 (17.3)	4 (5.3)	3 (4.0)
I feel confident that managing my warfarin therapy with a clinical pharmacist in a special clinic would improve my health	27 (36.0)	24 (32.0)	10 (13.3)	5 (6.7)	1 (1.3)
The clinical pharmacist can give me clear instructions about what dose of warfarin should I take	29 (38.7)	24 (32.0)	9 (12.0)	4 (5.3)	2 (2.7)
The clinical pharmacist can give me clear instructions about when to get my INR blood test done	29 (38.7)	25 (33.3)	12 (16.0)	2 (2.7)	1 (1.3)
I trust the clinic pharmacist to answer my questions about warfarin	34 (45.3)	17 (22.7)	15 (20.0)	2 (2.7)	1 (1.3)
Clinical pharmacists are well qualified to manage my warfarin if I experience side effects	28 (37.3)	19 (25.3)	17 (22.7)	4 (5.3)	1 (1.3)
I am confident that the clinical pharmacist can manage my warfarin treatment safely	27 (36.0)	22 (29.3)	16 (21.3)	3 (4.0)	1 (1.3)
My physician will easily refer me to the anticoagulation clinic if needed	34 (45.3)	27 (36.0)	5 (6.7)	2 (2.7)	1 (1.3)
The clinic pharmacist must work with other healthcare professionals to help manage my warfarin	28 (37.3)	32 (42.7)	6 (8.0)	3 (4.0)	0 (0.0)
Using the warfarin service managed by a clinical pharmacist means that the pharmacist is also able to help me with other aspects of my healthcare	25 (33.3)	21 (28.0)	17 (22.7)	4 (5.3)	2 (2.7)
The clinical pharmacist will help to decrease medication errors	29 (38.7)	19 (25.3)	18 (24.0)	3 (4.0)	0 (0.0)
I would prefer to have my warfarin managed by my physician rather than a clinical pharmacist	26 (34.7)	15 (20.0)	19 (25.3)	9 (12.0)	1 (1.3)

*The percentages shown in the results do not total 100%, as some participants chose not to answer certain questions

More than half of the respondents strongly agreed that implementing the clinic in primary care would benefit patients taking warfarin (50, 66.7%), improve their health (45, 60%), enhance adherence to medicine (40, 53.3%), provide more time to discuss their concerns (56%, n = 42), and result in easier appointment scheduling (50.7%, n = 38). Additionally, more than 40% (n = 33) of them strongly agreed that implementing the clinic in primary care would be more convenient than in hospital settings.

In the assessment of patients’ perspectives regarding anticoagulation monitoring services provided by a clinical pharmacist, more than one-third of the respondents (36.0%, n = 27) strongly agreed that the provision of care by a clinical pharmacist would improve their health. Most participants strongly agreed with the statement, indicating their confidence in the ability of clinical pharmacists to address their inquiries regarding warfarin (45.3%, n = 34). Similarly, the respondents expressed strong agreement with statements indicating their confidence in the clinical pharmacist’s ability to manage warfarin treatment safely (36.0%, n = 27) and decrease medication errors (38.7%, n = 29). The majority of participants strongly agreed that clinical pharmacists can provide clear instructions regarding the dose of warfarin and frequency of INR measurements (38.7%, n = 29). Most respondents strongly agreed that clinical pharmacists are highly qualified to handle warfarin treatment if they encounter side effects (37.3%, n = 28). More than 45% (n = 34) strongly agreed that their physician would easily refer them to the anticoagulation clinic if necessary. The majority (42.7%, n = 32) agreed that pharmacists should collaborate with other healthcare providers to effectively manage their therapy. Approximately 61% (n = 46) of patients agreed or strongly agreed that implementing this model would enable pharmacists to assist them with various other aspects of their healthcare. However, most patients (45.7%, n = 41) agreed that they preferred being managed by their physicians rather than by a pharmacist.

## Discussion

This study explored stakeholder perceptions about implementing pharmacist-led anticoagulation clinics in primary care in Qatar. Overall, most healthcare providers strongly supported the implementation of a pharmacist-led model in primary care, expecting efficient management, better health outcomes, and increased convenience compared to hospital settings. These results agree with previous studies that explored stakeholders’ perceptions toward providing anticoagulation services in primary care (Beyene et al., 2021; Egunsola et al., 2022; Rodgers et al., 1997; Shaw et al., 2014; Woodill & Bodnar, 2020).

However, although patients expressed confidence in clinical pharmacists’ ability to manage their medication therapy, many still preferred physicians’ management. These results are in contrast to a previous study that examined stakeholder attitudes toward community pharmacist-led anticoagulation management services in New Zealand, in which the majority of patients expressed a preference for pharmacist-led management (Shaw et al., 2014). The observed differences in results between the two studies can be attributed to the distinct settings, societal trust in the pharmacy profession and phases of implementation explored. The pre-implementation setting captures stakeholders’ expectations that may not fully align with the challenges or successes observed after implementation. Conversely, the post-implementation setting provides evidence of the model’s impact that may not have been anticipated in the planning phase (Aarons et al., 2011; Alley et al., 2023). Understanding these differences is essential for interpreting study findings and making informed decisions about model effectiveness in primary care.

Healthcare providers agree that pharmacist training is essential to effectively manage the clinic. Studies have shown that ensuring pharmacists’ clinical competencies is crucial for optimising service outcomes (Shaw et al., 2014; Tadesse et al., 2022). Successful implementations have involved a structured approach that includes formal education and ongoing professional development (Jebara et al., 2021; Shaw et al., 2014; Shimabukuro et al., 2004). The Anticoagulation Forum recommends that the anticoagulation management system must ensure appropriate staff training (Nutescu et al., 2013). Healthcare provider training typically begins with intensive education, covering the pharmacology of anticoagulants, the pathophysiology of thromboembolic disorders, and key principles of anticoagulation management (Nutescu et al., 2013). Clinical training is equally crucial, where pharmacists work under the guidance of experienced clinicians in anticoagulation clinics, applying their theoretical knowledge to real-world situations (Jebara et al., 2021). Case-based learning further enhances their decision-making and problem-solving skills through simulated scenarios (Shimabukuro et al., 2004). Additionally, training should emphasise interdisciplinary collaboration, fostering strong communication skills for effective coordination with other healthcare professionals (Nutescu et al., 2013). By integrating these elements, healthcare systems can ensure that pharmacists are well-prepared to manage anticoagulation clinics effectively.

Previous studies have highlighted suboptimal anticoagulation management in Qatar (Alshihab et al., 2024; Elewa & Ali, 2018). In this study, over one-fourth had experienced warfarin-related hospitalisation, and approximately half of the surveyed patients reported that warfarin monitoring significantly affected their daily activities. Moreover, a significant proportion of physicians indicated that they did not have confidence in managing patients on warfarin. Consistent with these findings, our prior qualitative investigation identified several factors contributing to this issue, including primary care physicians’ reluctance to manage patients on warfarin given their limited anticoagulation expertise, as well as unclear treatment plans for patients referred from secondary care. These findings underscore the pressing need for specialised clinics that can provide the expertise and resources needed for effective anticoagulation management (Raphael, 2020).

### Strengths and limitations

This study has several strengths. First, it explores the perceptions of key stakeholders at three different levels regarding the proposed model, offering a broad spectrum of perspectives that can inform future interventions and policies. In addition, the study was conducted within the largest primary care provider setting in Qatar, encompassing more than 30 centres nationwide.

However, it is important to acknowledge the limitations of this study. The study experienced low response rates for the distributed surveys, ranging from 10% to 48%, which could potentially impact the generalizability of the survey findings and underscore the need for cautious interpretation when considering the study results and their broader implications.

## Conclusions

Stakeholders expressed positive views about implementing a pharmacist-led anticoagulation clinic in primary care, demonstrating confidence in pharmacists’ ability to manage anticoagulation therapy effectively. This model could provide an efficient solution to address the current limitations in anticoagulation management.
